# Machine learning-based analysis of the relationship between brain lesion sites and swallowing and cognitive functions in stroke patients

**DOI:** 10.1038/s41598-026-52516-5

**Published:** 2026-05-14

**Authors:** Yusuke Hata, Seiji Hama, Akane Hamago, Shizuko Kiregawa, Yuki Sato, Ziqiang Xu, Akira Furui, Zu Soh, Harutoyo Hirano, Stephanie Sutoko, Tomohiko Mizuguchi, Akihiko Kandori, Takao Hamamoto, Sachio Takeno, Shigeto Yamawaki, Toshio Tsuji

**Affiliations:** 1 Graduate School of Advanced Science and Engineering, Hiroshima University, Higashihiroshima, Japan; 2 Hibino Hospital, Hiroshima, Japan; 3 Center for Brain, Mind and KANSEI Sciences Research, Hiroshima University, Hiroshima, Japan; 4 Department of Otorhinolaryngology, Head and Neck Surgery, Graduate School of Biomedical and Health Sciences, Hiroshima University, Hiroshima, Japan; 5 Fujita Health University, School of Medical Sciences, Toyoake, Japan; 6 New Business Producing Division, Business Development Department, Maxell, Ltd., Tokyo, Japan; 7 Healthcare Innovation Center, Research and Development Group, Hitachi, Ltd., Tokyo, Japan; 8 e-Health Business Development, Bwave Inc., Kanagawa, Japan; 9 Next Research, Research and Development Group, Hitachi, Ltd., Tokyo, Japan

**Keywords:** Dysphagia, Stroke, Cognitive function, Brain injury site, Machine learning analysis, Log-linearized Gaussian mixture network, Cognitive neuroscience, Diseases of the nervous system, Stroke

## Abstract

Stroke-related dysphagia is influenced by brain damage location and cognitive impairment, but its mechanisms remain unclear. In this study, we aimed to clarify the mechanisms by which brain damage causes dysphagia and cognitive function in 246 patients with stroke using a probabilistic neural network model. Dysphagia was classified as mild (oral intake with liquid and diet modifications) or severe (unable to take food orally, requiring tube feeding). Atlas-based segmentation applied to brain MRI data delineated 121 anatomically-defined regions, including 116 Automated Anatomical Labeling regions (AAL116), brainstem level 1, and white matter level 4 of the Automated Talairach Atlas Labels (ATAL), and the total score of five cognitive items on the Functional Independence Measure was used to evaluate cognitive function. Classifying dysphagia by severity and evaluating cognitive function resulted in improvements in prediction accuracy and reduced the number of predictor variables. In addition, after adding evaluations of cognitive function in both the severe and mild dysphagia groups, evaluations of the brainstem, which had remained a final predictor variable in the analysis of brain regions only, no longer remained. The results highlight the importance of integrating neurological imaging and cognitive assessment in the diagnosis and rehabilitation of dysphagia after stroke.

## Introduction

Stroke results from a vascular incident that actively damages specific areas of the brain. Depending on the location of the damage, symptoms affecting daily life and social reintegration, such as paralysis, aphasia, cognitive disturbances, and dysphagia, may occur^1^. Among these symptoms, dysphagia can lead to malnutrition and aspiration pneumonia, thereby increasing morbidity and mortality^2^. Therefore, early identification of dysphagia is crucial because timely recognition enables appropriate early stage management interventions (such as dietary modification and swallowing rehabilitation) to prevent complications associated with dysphagia.

Swallowing is an oral, oropharyngeal, and esophageal motor action that consists of voluntary and reflexive motions under the control of different levels of the central nervous system, from the cerebral cortex to the medulla oblongata^3^. To date, many studies have been conducted to elucidate the factors that contribute to the development of dysphagia from the point of view of the responsible lesion. However, the brain lesions that cause dysphagia remain controversial. Akahori et al.^4^ reported that damage to the cerebral cortex was associated with dysphagia, while de Alencar Nunes et al.^5^ reported an association between damage to the brainstem and dysphagia. They also found that white matter is associated with swallowing function and connects the swallowing center in the cortex with the central pattern generator (CPG) in the brainstem. The CPG controls swallowing patterns and receives modulatory input from the cerebral cortex through these white matter pathways. Impairment of this cortical modulation, which involves cognitive function, can cause dysphagia.

The connection between the CPG in the medulla oblongata and the swallowing center in the cortex can modulate swallowing depending on bolus properties such as texture and size^6^. Impairment of this modulation (the preparation before swallowing), which involves cognitive function, can cause dysphagia^7^, suggesting that cognitive function is involved in both the voluntary and reflexive components of swallowing. The relationship between dysphagia and cognitive impairment has been increasingly clarified in recent years, with several studies demonstrating a direct link between swallowing function and cognitive decline^8,9^. However, challenges remain in fully elucidating the underlying mechanisms, partly because patients with dysphagia are often excluded from cognitive studies due to coexisting aphasia or severe cognitive impairment, which complicates detailed assessments of executive functions^10^. Cognitive functions are supported by specialized neural networks within the brain, each of which is dedicated to a specific cognitive process^1^. This complexity makes it challenging to clarify the pathophysiology of swallowing disorders, where brain lesions and cognitive impairments are closely interconnected.

In the present study, we aimed to clarify the mechanisms, including cognitive function, by which brain damage causes dysphagia in patients with stroke using a probabilistic neural network model. The pathogenesis of dysphagia is complicated because various symptoms are intertwined with motor, sensory, and cognitive disorders, and variation exists with regard to the type and severity in the symptoms of dysphagia. Therefore, in this study, we classified dysphagia into two groups depending on severity: mild dysphagia, in which patients could intake food orally but required liquid and diet modification, and severe dysphagia, in which patients were not capable of oral intake and thus required tube-feeding. We investigated the hypothesis that assessing dysphagia classified by severity using machine learning techniques based on damaged brain regions and cognitive function could lead to a more targeted understanding of dysphagia.

## Methods

### Study design

This retrospective study was conducted under protocols approved by the Epidemiological Research Ethics Committee of Hiroshima University (under approval number E2018-1554-03) and the Ethics Review Committee of the Shinaikai Hibino Hospital. In accordance with the committee’s requirements, comprehensive informed consent for research participation was obtained from all subjects. However, due to the retrospective design of this specific analysis, the ethics committees waived the need for additional, study-specific informed consent. All methods were performed in accordance with the relevant guidelines and regulations of the Declaration of Helsinki.

### Participants

Data for this study were collected from stroke patients admitted to our convalescent rehabilitation ward between December 2019 and February 2024. During this period, our hospital admitted 899 stroke patients who were potentially eligible for inclusion. However, due to a shortage of testing personnel and the impact of the COVID-19 pandemic, 305 patients were tested. These patients were randomly selected from among all hospitalized patients. After applying the exclusion criteria detailed in the Participants section, our final sample consisted of 246 participants. This results in an inclusion rate of approximately 27% (246 out of 899) from the pool of all inpatients and approximately 81% (246 out of 305) when the impact of insufficient manpower and the pandemic is excluded. All subjects were orally ingesting food before the onset of stroke. Exclusion criteria included (1) history of major psychiatric illness, such as a major depression, bipolar disorder, schizophrenia, or schizoaffective disorder (1 patient); (2) history of neuromuscular disorders such as Parkinson’s disease (5 patients); (3) history of dementia such as Alzheimer’s disease (24 patients); (4) history of malignant tumor of the pharynx and larynx (2 patients); (5) Difficulty in analyzing the subject’s MRI images (poor image data, etc.) (6 patients) and (6) MRI could not be obtained within one month of admission to the convalescent rehabilitation ward (n=16). Subjects with moderate dysphagia (Gr. 4–6) were also excluded from the present analysis due to the insufficient sample size (n=5). Remaining 246 subjects were included in this study.

The period between the date of onset and the dates of admission and MRI acquisition were also calculated.

###  Assessment of cognitive function

The patients’ level of disability was assessed using the Functional Independence Measure (version 3.0), which is composed of 18 items (13 motor and 5 cognitive items) rated on a scale from 1 to 7 in terms of dependency (the lower the score, the greater the disability). The FIM is an observer-rating scale assessed at the time of admission and every few weeks by physical, occupational and speech and language therapists of rehabilitation, nurses and registered dietitians under the supervision of a board-certified rehabilitation physician in the convalescent rehabilitation ward. This study was conducted using medical records obtained in this manner.

To evaluate cognitive function in both the mild and severe dysphagia groups, we used the total score of the five cognitive items on the FIM (cFIM), a rating scale that can assess cognitive function even if the patient’s symptoms are so severe that a cognitive function test cannot be performed. Many patients with dysphagia in this study had significantly impaired physical and cognitive abilities, making it difficult to administer standardized cognitive assessments such as the Mini-Mental State Examination (MMSE) or Montreal Cognitive Assessment (MoCA). Therefore, the cFIM was selected as a practical and reliable alternative for assessing cognitive function in this population.

###  Lesion analysis

In addition, magnetic resonance imaging (Signa EXCITE, XI, ver. 11.0, GE Healthcare, Milwaukee, WI, USA) was performed to identify the damaged areas of the brain. Silent cerebral infarction and white matter hyperintensity (WMH) on MRI are known to contribute to declines in cognitive and physical functions^11^ and were therefore included in this study. However, distinguishing between these lesion types and previous stroke lesions using the image analysis methods employed in this study is challenging. Consequently, our brain image analysis encompassed symptomatic and asymptomatic old cerebral infarctions as well as WMH, recognizing the overlap and heterogeneity among these lesion etiologies. Cerebral hemorrhage can be effectively evaluated using the MRI gradient recalled echo (GRE) pulse sequence method (T2*), achieving an accuracy comparable to that of CT, depending on the time phase from symptom onset. However, this method is subject to overestimation in approximately 17.8% of cerebral hemorrhage cases^12,13^. In contrast, while cerebral hemorrhage is optimally visualized with GRE, the evaluation of cerebral infarction and associated pathologies benefits from complementary sequences. FLAIR and DWI imaging are more sensitive for detecting acute-to-subacute ischemic changes and are also valuable for identifying the peripheral edema surrounding cerebral hemorrhage, as well as for detecting concomitant cerebral infarction and white matter hyperintensity (WMH)^14^. Therefore, cerebral hemorrhage evaluated using GRE and DWI was checked as a lesion on FLAIR images together with cerebral infarction (if present) and WMH. Therefore, Diffusion-weighted imaging, fluid-attenuated inversion recovery, T2*, T1-weighted imaging, and T2-weighted imaging sequences were obtained from the brain. The following five steps were used to quantify the damaged areas of the brain.

**Fig. 1:**
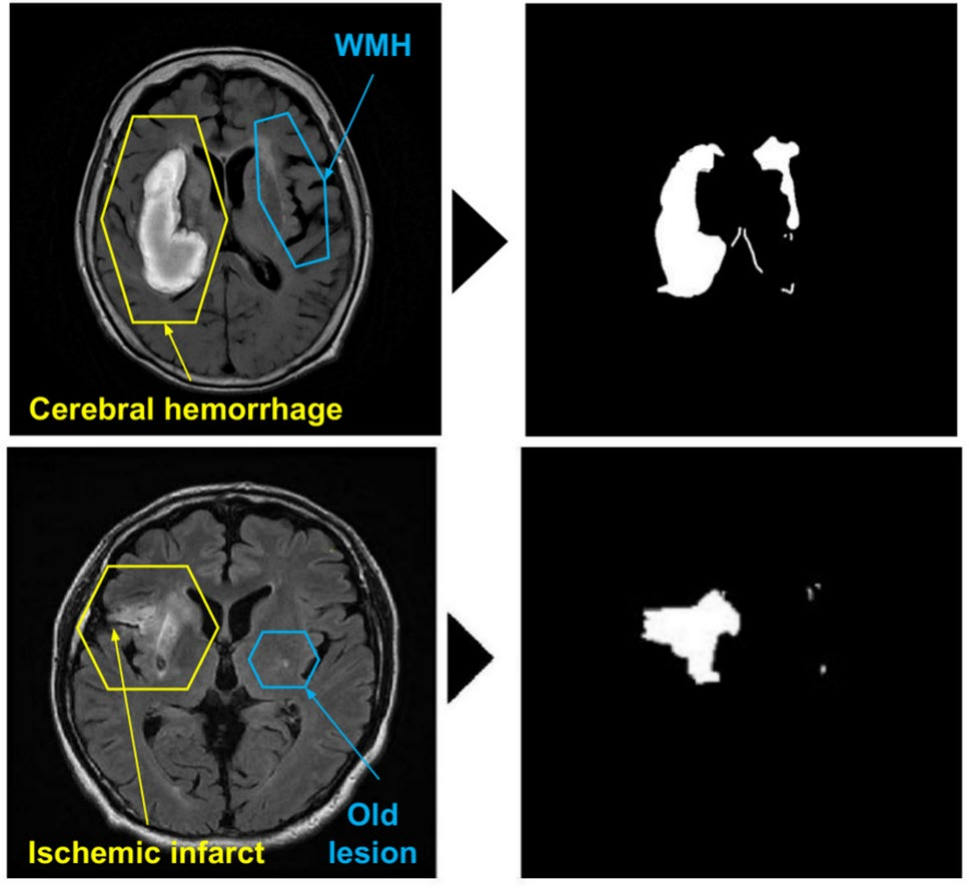
Manual Lesion Segmentation Process from T2-FLAIR Images. Illustration of the lesion extraction process in a patient with intracerebral hemorrhage (top row) and ischemic infarct (bottom row). (Left) Original axial T2-FLAIR image. Manual lesion masking was performed by a specialist to ensure clinical accuracy. Hematomas or cerebral infarcts are outlined in yellow hexagons, while old lesions or white matter hyperintensities (WMH) are outlined in light blue. (Right) Final extracted lesion mask used for the quantitative analysis.

1. The raw data format – Digital Imaging and Communications in Medicine (DICOM), was converted to the format of Neuroimaging Informatics Technology Initiative (NIfTI). Images depicted in 3D intensity arrays were then segmented for different tissue types, including gray matter, white matter, cerebrospinal fluid, skull, and soft tissue. Pixels with zero probability for all tissue types were considered the background pixels. Steps of DICOM conversion and tissue segmentation were done using SPM12 (The Wellcome Centre for Human NeuroImaging, University College London, London, United Kingdom) prior to the lesion segmentation process.
2. A neurosurgeon identified and manually labeled the injury site using MRIcron^1,15–17^.
3. Brain images were normalized (nonlinear spatial normalization)^15,18^ and standardized between patients according to the Montreal Neurological Institute brain template (2 × 2 × 2 mm voxel size) (Fig. 1).
4. In addition to automated anatomical labeling (AAL116)^19–21^, brain regions were labeled according to the left and right parts of the brainstem in level 1 of Automated Talairach atlas labels (ATAL)^22^, and the left and right parts of the white matter, as well as “* white matter” (labeled for regions with inter-hemispheric or unspecified labels at Level 1), in level 4 of ATAL (total of 121 brain regions).
5. For each labeled region, the number of voxels in the damaged area and the total number of voxels were calculated.
6. For each region, the degree of brain damage was calculated from the ratio of the number of voxels in the damaged area to the total number of voxels.

###  Lesion overlay mapping

**Fig. 2:**
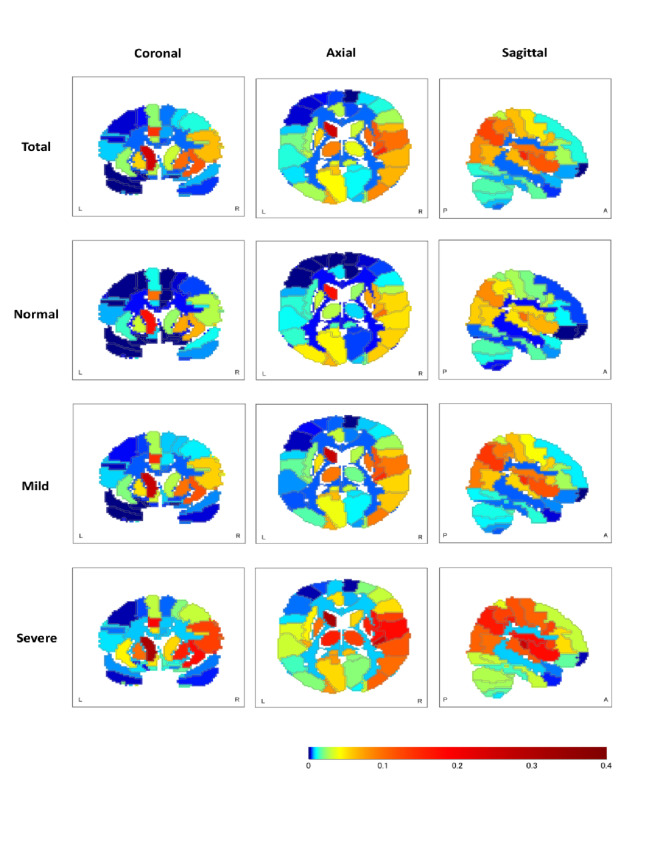
Mean lesion distribution across AAL regions. The image shows axial, coronal, and sagittal slices of the brain with color-coded mean lesion frequencies for each AAL region, aggregated across all cases. The colors in the figure represent the mean ratio of lesion volume to total volume for each region in the corresponding cases.

To provide a visual representation of lesion distribution across anatomical regions, we created a lesion overlay map based on the Automated Anatomical Labeling (AAL) atlas. For each AAL region, we calculated the mean lesion frequency across all included cases. These values were then projected onto a standard brain template to generate a representative image of lesion distribution. This visualization was included to facilitate assessment of the spatial characteristics of the lesions in relation to the AAL-defined brain regions (Fig. 2).

###  Assessment of swallowing function

The following outlines the diagnostic and rehabilitation process for dysphagia at our hospital.

1. Patients are first evaluated using the Seirei dysphagia screening questionnaire^23^. If the screening indicates possible dysphagia, a multidisciplinary team (board-certified rehabilitation physician, nurse, speech and language therapists of rehabilitation and registered dietitians. And if necessary, Otolaryngologist) becomes involved.
2. Newly admitted patients underwent a modified Water Swallow Test^24^. For patients who were transferred, previous hospital medical records, including videofluoroscopic swallowing study (VFSS) results, were reviewed, and additional tests (modified Water Swallow Test, FEES, and/or VFSS) were performed if clinically indicated.
3. If dysphagia is clinically indicated, fiberoptic endoscopic evaluation of swallowing (FEES) is performed early to assess swallowing function in detail. The hyodo score and Penetration aspiration score (PAS) are used to assess swallowing function during FEES^25,26^.
4. Based on test results, the severity of dysphagia is determined, and a rehabilitation approach is planned.

The data for this study were collected at the stage 2 to 4 above, when patients were transferred from our general ward providing acute care or from other hospitals, and admitted to our convalescent rehabilitation ward.

The participants’ swallowing function scores used in this analysis were evaluated using the Food Intake LEVEL Scale^27^, which evaluates the severity of eating and swallowing disorders on a 10-point scale. The FILS is assessed at the time of both admission and discharge by speech and language therapists of rehabilitation, certified nurse of dysphagia nursing and registered dietitians under the supervision of a board-certified rehabilitation physician in the nutrition support team certified by Japanese Society for Parenteral and Enteral Nutrition Therapy (JSPEN) of the convalescent rehabilitation ward. In total, 126 participants were classified as having mild dysphagia (Gr. 7–8: oral intake alone with some support, i.e., easy-to-swallow food or consideration of some medical issues), 48 as severe (Gr. 1–3: no oral intake and require tube-feeding or intravenous drip for nutrition), and 72 as non-dysphagic controls without dysphagia (Gr. 10). Nasogastric tube feeding was performed in patients who had difficulty in oral intake due to severe dysphagia confirmed by VFSS (n=21) or FEES (n=2), in patients who had persistent impaired consciousness and/or who produced a large amount of sputum and required frequent suctioning procedures, making it difficult to perform a swallowing study (n=23), or no chewing or feeding movement into the pharynx was induced even when food was placed in the mouth (n=2).

In this study, patients classified as FILS level 9 were excluded, as communal dining was restricted due to COVID-19 precautions, resulting in very few cases at this level. Therefore, mild cases were defined as those at FILS levels 7 and 8.

In this study, the number of subjects with FILS levels 4 to 6 was too small to be included in the analysis. The reason is that stroke patients are often transferred to the Convalescent Rehabilitation Ward of our hospital immediately after acute care, combined use of enteral and oral nutrition is uncommon. Even when both are used, signs of aspiration—such as coughing during meals, pneumonia on imaging, or elevated inflammatory markers—often lead to exclusive enteral feeding. As a result, patients classified as FILS levels 4 to 6, who receive both enteral and oral nutrition, are relatively few. However, early swallowing evaluations using FEES or VFSS are routinely performed to guide rehabilitation planning. In accordance with the 2021 classification by the Japanese Society of Dysphagia Rehabilitation^28^, we prepared test foods corresponding to each code: Code 0j (jelly), Code 1j (when necessary), Code 2 (2-1 and 2-2), Code 3, and Code 4. These included adjusted staple foods (rice porridge, rice) and side dishes. We also included regular consistency foods. For liquids, we used non-thickened (thin liquid) and three thickened stages: mildly thick, moderately thick (Stage 2), and extremely thick (Stage 3).

###  Statistical analysis

To compare differences between three groups (control, mild dysphagia and severe dysphagia groups), $\chi ^2$ test was used for categorical variables and the Kruskal-Wallis test followed by the Steel-Dwass test was used for quantitative variables, with JMP Student Edition 18.0 (SAS Institute Inc., Cary, NC). The level of significance was set at p < 0.05.

### Dysphagia identification model

**Fig. 3:**
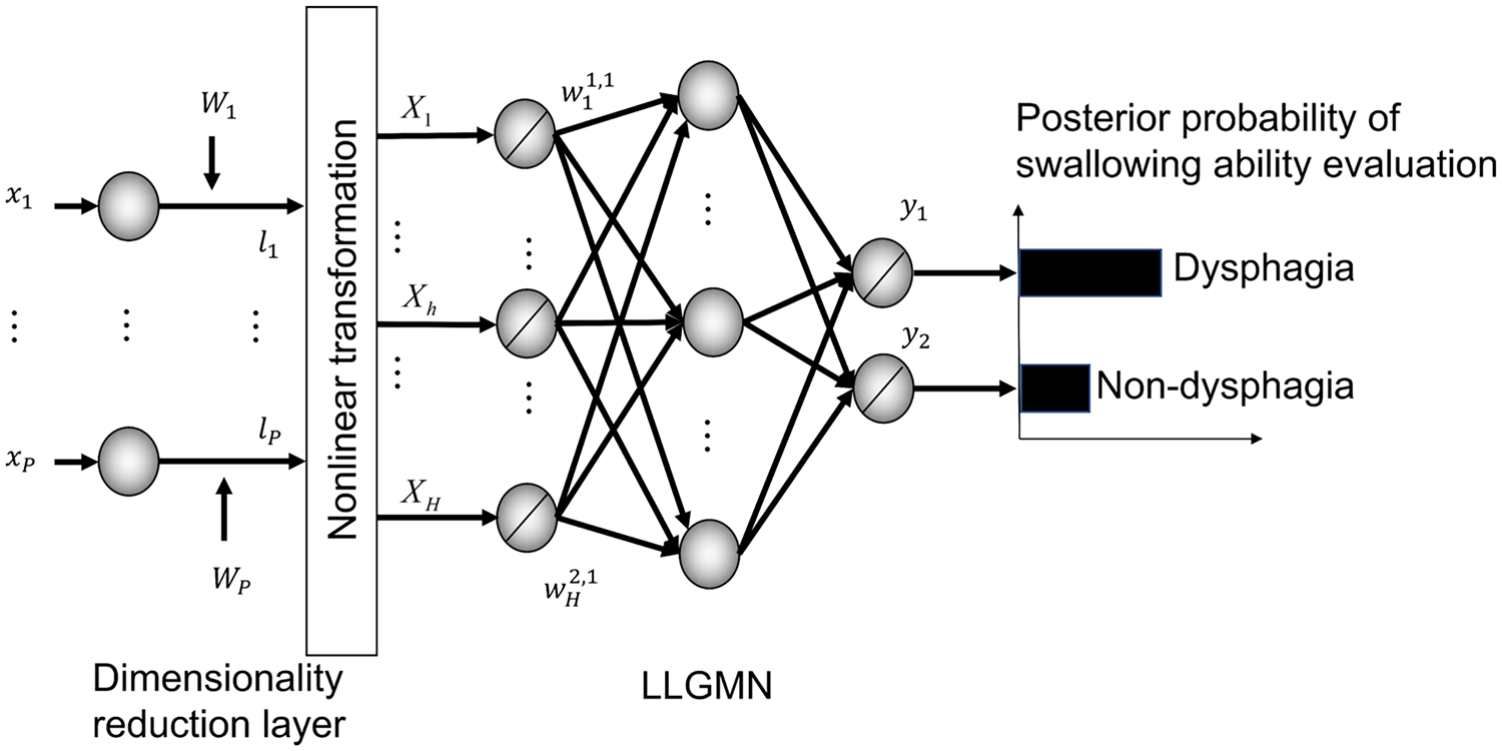
Overview of the discriminant model of dysphagia based on an LLGMN.

To analyze the relationship between brain damage locations and swallowing function assessment, machine learning was performed using a log-linearized Gaussian mixture network (LLGMN)^29^, a type of probabilistic neural network that includes the mixed normal distribution. An LLGMN can estimate the statistical distribution followed by sample data in a machine learning manner and estimate the posterior probability of the class to which the unknown input data belong. In this study, we estimated the posterior probability of dysphagia. However, an LLGMN alone cannot clarify the relationship between the degree of damage in each brain region and the assessment of swallowing function for the features of the cFIM score at admission. Figure 3 shows a schematic diagram of the LLGMN with a dimensionality reduction layer. The input is a *P*-dimensional index value ${\textbf{x}}={[x_1, x_2,..., x_P]}^T\in \mathbb {R}^P$, and the output is a two-dimensional posterior probability vector ${\textbf{y}}={[y_1, y_2]}^T\in \mathbb {R}^2$ representing the groups of patients with and without dysphagia. The relationship between the inputs and outputs in the dimensionality reduction layer can be expressed by the following equation:

1 $$\begin{aligned} l_i= & W_i x_i \;\;(i=1,2,...,P) \end{aligned}$$

where $l_i$ is the output of the dimensionality reduction layer and $W_i$ are the weights of the dimensionality reduction layer. The LLGMN dimensionality reduction layer used in this study can automatically reduce the $W_i$ of input features unrelated to the output to near 0 at the end of training by applying $L_1$ regularization and $L_2$ regularization to the dimensionality reduction layer weights $W_i$. By excluding these input features, it is possible to extract only those input features relevant to dysphagia.

Using the index value $\textbf{x}^{(n)}(n=1,2,... N:$ N is the number of subjects used in the training) and the teacher data $\boldsymbol{Q}^{(n)}=[Q_1^{(n)},Q_2^{(n)}]^T\in \mathbb {R}^2$ indicating the presence or absence of swallowing function, the LLGMN dimensionality reduction is trained. The loss function *E* of the LLGMN dimensionality reduction layer is defined by the following equation:

2 $$\begin{aligned} E= & -\sum _{n=1}^{N} \sum _{k=1}^{2}\alpha Q_k^{(n)}\log {y_k^{(n)}} +\lambda _1\sum _{i=1}^{P}|W_i|+\lambda _2\sum _{i=1}^P|W_i|^2 \end{aligned}$$

where $\lambda _1$ is the $L_1$ regularization and $\lambda _2$ is the regularization parameter that determines the strength of the effect of the $L_2$ regularization. The first term in Equation (2) represents the weighted cross-entropy error. The second term is the $L_1$ regularization term, which penalizes the error according to the magnitude of the absolute value of the weights $W_i$ in the dimension reduction layer. The third term is the $L_2$ regularization term, which penalizes the error according to the square of the magnitude of the absolute value of the weights $W_i$ in the dimensionality reduction layer. The $\alpha$ coefficient takes the imbalance in the number of data sets between the classes of dysphagia ratings used for training into account, and is represented by the following equation:

3 $$\begin{aligned} \alpha= & \left\{ \begin{array}{l} \frac{N}{2n_0}\;\text {if}\, {Q^{(n)}}=0 (non-dysphagic patients) \\ \frac{N}{2n_1}\;\text {if}\, {Q^{(n)}}=1 (dysphagic patients) \end{array} \right. \end{aligned}$$

where $n_0$ represents the number of non-swallowing patients and $n_1$ represents the number of patients with dysphagia. The $\alpha$ coefficient can be multiplied by the first term to penalize the number of records in the class between the presence and absence of dysphagia according to the bias in the number of records. By minimizing the loss function E based on the error backpropagation method, the parameters of the entire network can be learned to reduce the error as long as $W_i$ does not become large. A cost-weighted learning approach using the $\alpha$ coefficient was implemented, where $\alpha$ was calculated based on class imbalance to automatically penalize the misclassification of minority classes according to the formula presented above.

### Analysis method

In this study, we analyzed (i) a group of patients with mild dysphagia, (ii) a group of patients with severe dysphagia, and (i) a group of patients with any type of dysphagia (mild + severe) against a group of patients without dysphagia as a control. The association between dysphagia, damaged brain regions, and cognitive function was analyzed using the following inputs (a) and (b).

- Degree of damage in each brain area (121 areas) (brain area)
- 121 combinations of brain area damage and total cFIM score on admission (brain area + cFIM score on admission)

Machine learning with an LLGMN with dimensionality reduction was used to select indicators to assess dysphagia when the presence of dysphagia was used as a supervised signal and (a) and (b) above were used as input indicators. This methodological rigor ensured the separation of the training and validation stages, prevented information leakage, and guaranteed that the reported performance metrics reflected the true generalization capability of the independent test data, thereby addressing class imbalance while maintaining robust and reproducible model performance across the three dysphagia categories. The selection of valid indicators for each type of dysphagia was performed by stratified fivefold cross-validation (5-fold CV), and input indicators with an absolute mean weight $W_i$ of less than 0.01 after learning with the initial values of weights $W_i$ in the dimension reduction layer all set to 1 were excluded. The 5-fold CV was then performed again with only the selected input features, and the discrimination accuracy and confusion matrix were obtained based on the posterior probabilities and teacher signal. The magnitude of the sparsified weights $W_i$ was also checked to investigate the relationship between the swallowing function assessment and the degree of damage in each brain region. The LLGMN was trained using the stochastic gradient descent (SGD) method, with the SGD learning rate set to 0.01, the batch size equal to the split data size, and the number of epochs set to 10,000. Training was terminated when the loss on the training data did not improve by more than 0.0001 for more than five epochs. The number of LLGMN components was set to 1^30^. The tree-structured Parzen Estimator^31,32^ was used to optimize the regularization parameters of the LLGMN dimensionality reduction layer, and $\lambda _1$ and $\lambda _2$ were used to obtain the maximum discrimination accuracy. The analysis was performed 10 times to account for differences due to the initial values of the regularization parameters.

The model’s robustness against overfitting was ensured through three complementary mechanisms: (i) automatic weight compression of irrelevant features via $L_1$ and $L_2$ regularization, (ii) cost-weighted learning to address class imbalance, and (iii) nested stratified cross-validation with a strict separation of training and validation data. Together, these mechanisms prevented both model overcomplication and data leakage.

**Table 1:** Clinical characteristics of the patients with dysphagia in this study.

	Control group	Mild dysphagia group	Severe dysphagia group	*p*-value
	($n = 72$)	($n = 126$)	($n = 48$)	
Age (years)	$68.3 \pm 11.6 (73)$	$75.0 \pm 11.0 (77)$	$75.8 \pm 11.0 (78.5)$	< 0.0001
Sex (male), *n* (%)	52 (72.2[%])	79 (62.7[%])	23 (47.9[%])	0.0264
Past history of stroke, *n*(%)	9 (12.7[%])	22 (18.6[%])	10 (19.2[%])	0.424
Period between onset and admission (days)	$10.8 \pm 6.8 (11)$	$15.3 \pm 10.8 (14)$	$17.9 \pm 12.2 (14)$	0.0047
Period between onset and MRI (days)	$9.6 \pm 9.9 (6)$	$16.2 \pm 15.3 (10)$	$20.3 \pm 19.9 (13.5)$	0.0021
Disease				
Ischemic infarct, *n* (%)	47 (65.3[%])	74 (58.7[%])	21 (43.8[%])	0.0615
Hemorrhage, *n* (%)	25 (34.7[%])	52 (41.3[%])	27 (56.3[%])	
Stroke severity				
cFIM score on admission	$27.3 \pm 6.9 (29.5)$	$19.1 \pm 6.9 (19)$	$8.9 \pm 5.8 (7)$	< 0.0001

The table shows differences between the control and dysphagia (mild, severe) groups. All results except those for brain regions are presented as the mean ± SD (median), or number(%). The results for brain regions are presented as the mean ± SD in percentages. *p*-values are indicated using the $\chi ^2$ test for categorical values and the Kruskal–Wallis test for continuous values. Significant *p*-values (< 0.05) are in bold. cFIM: cognitive Functional Independence Measure.

## Results

### Participant characteristics

The findings in the present study that cognitive dysfunction, aging, and the presence of hemorrhage were associated with the presence and/or severity of dysphagia were consistent with those in previous reports^8,33^. Table 1 shows the backgrounds of the patients in the input data set used in this study. Significant differences in cFIM scores and age were found between the three groups of patients classified by the degree of dysphagia symptoms. The patients in the severe dysphagia group had lower cFIM scores, were older, and had higher rates of hemorrhage than those in the mild dysphagia and control groups. Similarly, the period between stroke onset and admission was significantly longer in the mild (p = 0.0258) and severe (p = 0.0071) dysphagia groups compared to the control group. A similar trend was observed for the period between onset and MRI acquisition, with significant differences between the control group and both the mild (p = 0.009) and severe (p = 0.0066) dysphagia groups. However, no significant differences were observed in these intervals between the mild and severe dysphagia groups (p=0.5338).

### Estimation accuracy (Figure 4, Table 2)

**Fig. 4:**
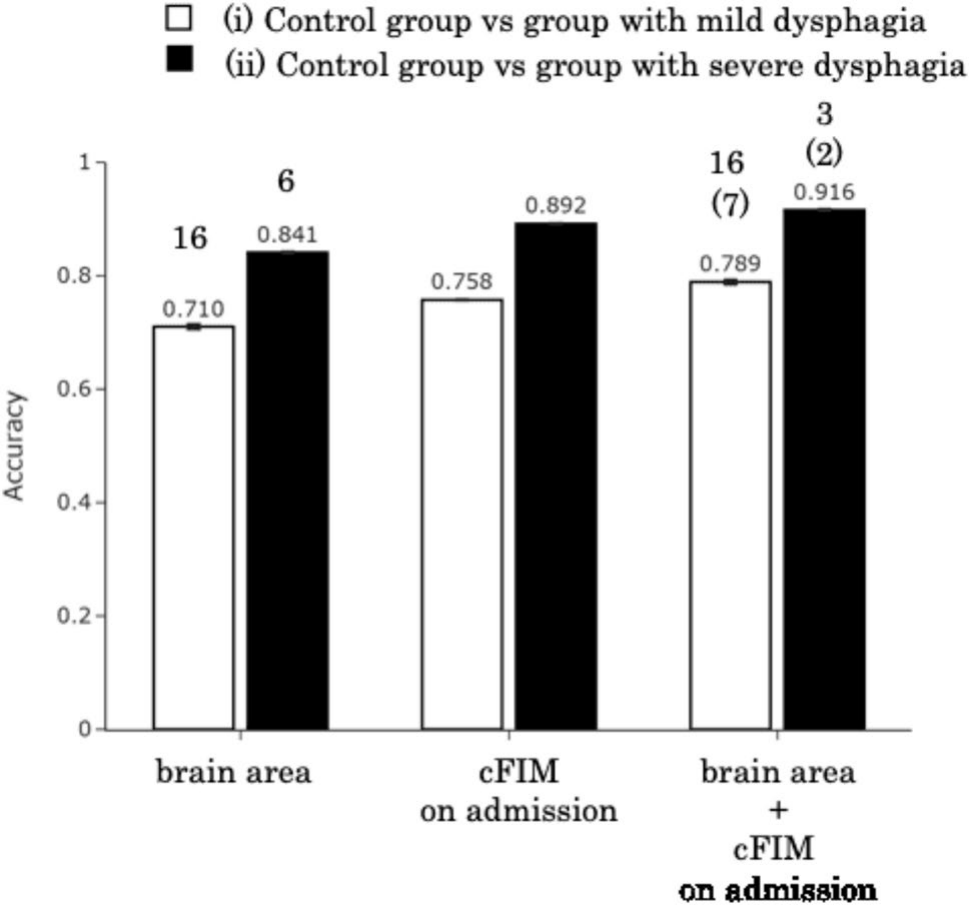
Identification results between the control and dysphagia groups.

Estimation accuracy is visually summarized in Figure 4, and detailed numerical values are provided in Table 2.

- For mild dysphagia, accuracy improved from 0.71 (brain area only) to 0.789 with the addition of cFIM.
- For severe dysphagia, accuracy increased from 0.841 to 0.916 when cFIM was included.

These results demonstrate that incorporating cFIM scores significantly enhances model performance across both severity levels.

### Selected indicators and brain regions in mild dysphagia models (Figure 5)

**Fig. 5:**
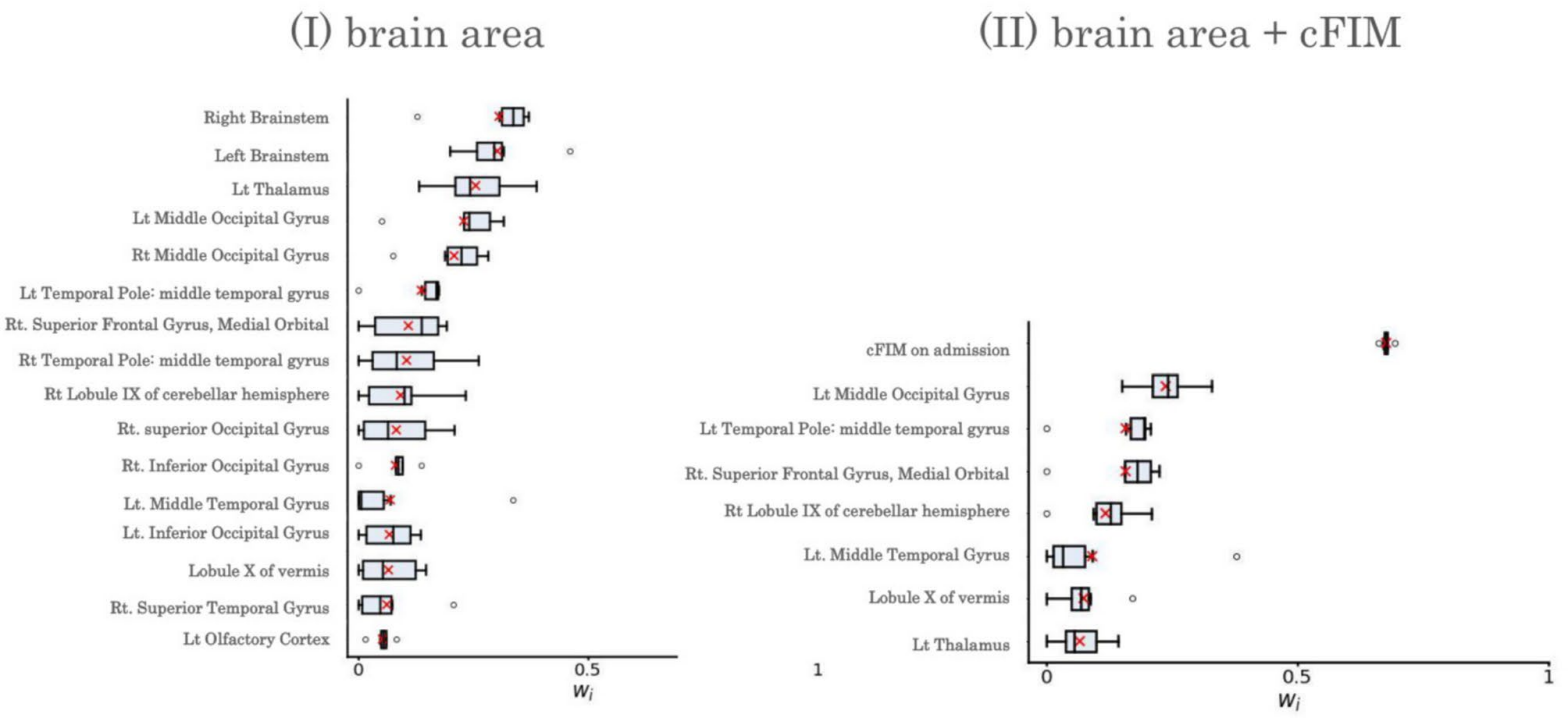
Optimized weights $W_i$ of (I) the brain damage areas and (II) cFIM scores on admission, as well as brain damage area indices selected by analysis (i): ‘Control group vs. group with mild dysphagia’. Box plots show median, quartiles, and 10th and 90th percentiles. The red crosses indicate the mean values.

Figure 5 shows the indicators selected for mild dysphagia.

- In the brain area only model (Figure 5-I), 16 brain regions were detected, with the brainstem appearing as a high-ranking indicator alongside regions in the cerebrum and cerebellum.
- In the brain area + cFIM model (Figure 5-II), the number of detected regions was reduced to 7, and the brainstem disappeared from the indicators. The cFIM score emerged as the most influential predictor variable, showing the highest mean weight ($W_i$) among all indicators.

These findings suggest that cognitive and functional status plays a dominant role in identifying mild dysphagia and helps refine the model by focusing on fewer, more relevant brain regions.

### Selected indicators and brain regions in severe dysphagia models (Figure 6)

**Fig. 6:**
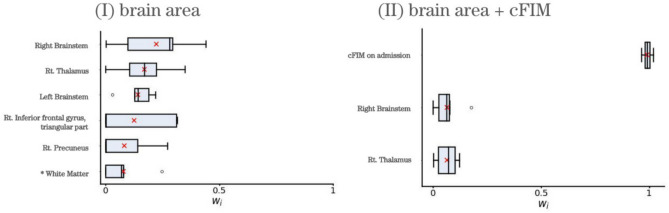
Optimized weights $W_i$ of (I) the brain damage areas and (II) cFIM scores on admission, as well as brain damage area indices selected by analysis (ii): ‘Control group vs. group with severe dysphagia’. Box plots show median, quartiles, and 10th and 90th percentiles. The red crosses indicate the mean values. * White Matter is labelled for regions with inter-hemispheric or unspecified labels in level 1.

Figure 6 presents the indicators selected for severe dysphagia.

- In the brain area only model (Figure 6-I), 6 brain regions were detected, with the brainstem again appearing as a prominent indicator.
- In the brain area + cFIM model (Figure 6-II), the number of detected regions was reduced to 2, yet the brainstem remained among the selected indicators. The cFIM score showed the highest mean weight ($W_i$), indicating its strong influence in the model.

These results highlight that, while the brainstem remains critical in severe dysphagia, cognitive function—as captured by the cFIM score—is the most influential predictor variable in both mild and severe cases.

Figure 4 shows the identification results for the control and dysphagia groups. The numerical values above each bar indicate the overall classification accuracy, expressed as decimal values ranging from 0 to 1, achieved by the LLGMN model for each analytical scenario. Panel (i) illustrates the comparison between the control and mild dysphagia groups, whereas panel (ii) depicts the comparison between the control and severe dysphagia groups. For each dysphagia category, two accuracy values were provided: one based solely on brain area data and the other incorporating both brain area data and the cFIM score upon admission. Detailed numerical values, including sensitivity, specificity, positive and negative predictive values, and kappa coefficients, are available in Table 2.

**Table 2:** Performance of classification ability between analyses (i)–(ii) by the proposed method compared with those based on diagnoses by medical doctors.

	Analysis (i): Control group vs. mild dysphagia		Analysis (ii): Control group vs. severe dysphagia	
Input Brain area	Diagnostic results by the proposed method		Diagnostic results by the proposed method	
	Subjects with dysphagia	Subjects without dysphagia	Subjects with dysphagia	Subjects without dysphagia
Classified results by medical doctors	50.1	21.9	60.7	11.3
	35.5	90.5	7.8	40.2
Subjects with dysphagia	PPV: 50.1%	NPV: 79.8%	PPV: 84.3%	NPV: 83.8%
	Sensitivity: 58.5%	Specificity: 80.5%	Sensitivity: 88.6%	Specificity: 78.1%
Subjects without dysphagia	Overall accuracy: 71.0%	Kappa coefficient: 0.40	Overall accuracy: 84.1%	Kappa coefficient: 0.67
Input Brain area + cFIM score	Diagnostic results by the proposed method		Diagnostic results by the proposed method	
	Subjects with dysphagia	Subjects without dysphagia	Subjects with dysphagia	Subjects without dysphagia
Classified results by medical doctors	58.8	13.2	64.1	7.9
	28.6	97.4	2.2	45.8
Subjects with dysphagia	PPV: 81.7%	NPV: 77.3%	PPV: 89.0%	NPV: 95.4%
	Sensitivity: 67.3%	Specificity: 88.1%	Sensitivity: 96.7%	Specificity: 85.3%
Subjects without dysphagia	Overall accuracy: 78.9%	Kappa coefficient: 0.56	Overall accuracy: 91.6%	Kappa coefficient: 0.83

## Discussion

This study aimed to clarify the mechanisms by which brain damage causes dysphagia in stroke patients using machine learning analysis. By separately analyzing mild and severe dysphagia, we gained clearer insights into the brain regions and cognitive function associated with each severity level. This stratified approach revealed both shared and distinct neural mechanisms, which may have been less apparent in a combined analysis. As the lesions responsible for dysphagia, the cerebral cortex, thalamus, and brainstem were selected as the indicators with the greatest influence on dysphagia identification on admission; this finding is consistent with previous studies reporting that these brain regions are associated with dysphagia^4,34–38^.The neural control mechanism of swallowing usually consists of the following three components: (1) peripheral input transmitted to the swallowing center via the trigeminal nerve^34^, (2) the swallowing center in the brainstem, which generates firing patterns and coordinates muscle activity^34^, and (3) the frontal cortex, which initiates and modifies swallowing^35^. Signal transmission between (1) and (2) passes through the neural pathway connecting the sensory receptors, trigeminal nerve, and swallowing center^34^. In addition, during signal transmission between (2) and (3), a closed circuit is formed connecting the cortex, white matter, thalamus, brainstem, and medullary swallowing center^35,36,39^. Damage to even one of these elements may increase the incidence of dysphagia. It has also been reported that damage to the cerebellum can cause dysphagia^37^, and that motor and sensory control of the cerebellum affects swallowing behavior^38^. The cerebellum receives inputs from the motor and sensory cortices and fine-tunes these inputs to control motor output. In this process, signal transmission is thought to take place in a circuit that connects the motor cortex, thalamus, and cerebellum. Thus, a wide range of brain regions is involved in swallowing, which is consistent with the present results showing that an analysis of swallowing disorders as a whole could not reveal the responsible lesion.

There is no doubt that damage to the swallowing center in the brainstem can cause dysphagia. However, in the model using both ‘brain regions’ and ‘cFIM scores’, cFIM scores showed the strongest correlation, and the cerebral cortex, basal ganglia, and cerebellum related to cognitive function were selected as the related brain regions. This suggests that cognitive function and the related brain regions are important for the prediction of swallowing disorders.

In contrast, mild dysphagia presents different challenges. The discrimination accuracy of the models predicting dysphagia was lower for mild cases than for severe cases, and the responsible lesions differed. This differential pattern suggests that the pathology of dysphagia may differ between mild and severe cases. For severe dysphagia, our stratified analysis revealed a meaningful relationship between brain regions (particularly those supporting cognitive and executive functions), functional status (cFIM scores), and the severity of swallowing impairment. The cerebral cortex, basal ganglia, and cerebellum emerged as key associated regions, along with brainstem involvement. These findings suggest that cognitive function and related brain regions are strongly associated with severe dysphagia and may support early risk identification when specialized assessments are unavailable. However, the situation is substantially different for mild dysphagia. The significantly lower prediction accuracy indicates that the underlying mechanisms remain incompletely understood, with multiple unidentified predictor variables likely influencing the presentation. The lower accuracy may be partly attributable to the limited range of severity in our dataset (FILS level 9 was excluded), and our inability to fully assess pre-stroke dietary modifications in this retrospective study may have introduced confounding factors in our results. Therefore, while our findings for severe dysphagia may have clinical utility, we acknowledge that conclusions regarding mild dysphagia remain preliminary and require further investigation with larger sample sizes and more detailed cognitive assessment protocols in the future.

Although the cFIM does not assess specific cognitive or executive functions, it reflects the overall functional status influenced by these abilities. In this study, we employed cFIM as a general indicator of cognitive function because many patients with dysphagia find it difficult to undergo detailed neuropsychological testing because of their suffering of severe impairments. Consequently, discussing a direct causal relationship between specific cognitive decline and dysphagia is methodologically challenging. Furthermore, because the cFIM encompasses a broad range of cognitive functions, it necessarily includes some domains that may not have direct mechanistic links to post-stroke swallowing control. Given these considerations, rather than a direct causal relationship, the association between cognitive impairment and dysphagia is more accurately understood as a manifestation of common neuropathological factors induced by stroke itself. Specifically, lesion sites previously reported to be associated with dysphagia, such as the cerebral cortex and thalamus^34–36^, simultaneously contribute to cognitive impairments. These anatomically shared substrates suggest that both dysphagia and cognitive deficits may arise concomitantly from a common lesion rather than through a primary cognitive mechanism that causes swallowing dysfunction. Additionally, the severity and extent of the stroke lesion influence the severity of both conditions, indicating that stroke severity and lesion size should be considered important background factors in future predictive models. Nevertheless, consistent with previous literature documenting the involvement of cognitive decline in dysphagia, our findings demonstrate that even a general cognitive score, rather than assessments of specific cognitive domains, shows a strong predictive value for dysphagia when combined with neuroimaging data identifying lesion location. This suggests that combining broad functional cognitive assessment with MRI-based lesion analysis may facilitate dysphagia risk identification without requiring extensive specialized testing, which has important implications in clinical rehabilitation settings.

## Limitations

This study has several limitations. First, in this study, we excluded subjects with major underlying conditions known to cause swallowing disorders, such as neurological diseases (e.g., Parkinson’s disease), dementia, and psychiatric disorders. However, due to the limited number of subjects, we were unable to analyze the individual effects of medications and comorbidities that may also contribute to dysphagia. In future research, we aim to increase the number of cases to enable a more detailed analysis of these factors.

Second, our study cohort was recruited during the rehabilitation phase after acute-phase stroke treatment was completed, resulting in heterogeneous intervals between stroke onset and MRI acquisition across the subjects. We analyzed brain lesions that included not only new cerebral hemorrhage and cerebral infarction, but also white matter hyperintensity (WMH), old cerebral hemorrhage, and cerebral infarction. After several months to years, structural and functional reorganization of the nervous system, known as neuroplasticity, occurs, which also causes morphological changes in the brain (e.g., encephalomalacia)^1^. Therefore, the accuracy of image reconstruction on a normal brain template may be lower than that in earlier studies. Additionally, we found a significant difference in the interval between stroke onset and MRI acquisition when comparing the control group to the mild and severe dysphagia groups, although no significant difference was found between the mild and severe dysphagia groups. This temporal heterogeneity, combined with neuroplasticity-induced structural changes, may influence the accuracy of lesion mapping and outcome predictions. Therefore, careful adaptation of brain image analysis, including old cerebral infarction and cerebral hemorrhage, is required. Future studies employing standardized MRI acquisition timelines relative to stroke onset (e.g., acute, subacute, and chronic phases) should clarify the independent effects of acute versus chronic lesions on swallowing and cognitive function.

Third, we were unable to examine whether the oral diet before the onset of stroke was modified, which may affect the estimation of mild dysphagia. More detailed studies are needed that take into account the influence of the status of the oral diet before the onset of stroke. Machine learning used in this study is believed to have shown potential as a method for this future study.

Fourth, cognitive function was assessed solely using the cognitive domain of the FIM (cFIM), which, while practical for patients with severe impairments, does not provide detailed information on specific cognitive domains. In future studies, we plan to incorporate digital devices or alternative assessment tools to enable more precise evaluation of cognitive function and investigate its relationship with individual swallowing impairments.

Fifth, due to the limited sample size, this study was unable to examine in detail the relationships between individual components of the feeding and swallowing process and the associated cognitive functions or brain regions. Further research with a larger cohort is needed to clarify these associations.

Sixth, this study has limitations regarding its generalizability. The data were collected from a single institution, and the patient recruitment period (December 2019 to February 2024) coincided with the COVID-19 pandemic. Future multicenter studies are necessary to validate our results in a more diverse cohort.

The seventh limitation concerns the comprehensive assessment of swallowing function. Ideally, all patients should undergo instrumental evaluations, such as VFSS or FEES, to obtain an accurate and definitive assessment of swallowing physiology. However, because these procedures are invasive and carry inherent risks and burdens for patients, their use in clinical practice is restricted to cases in which testing is clinically indicated and medically safe. Consequently, reliance on clinical observation rather than universal instrumental assessment may have influenced the accuracy of dysphagia classification in this study. Nevertheless, it is important to note that patients with dysphagia underwent rehabilitation with periodic instrumental evaluations using FEES or VFSS as their clinical condition evolved, allowing for objective physiological assessment to guide rehabilitation intervention.

## Conclusions

By separating dysphagia into mild and severe categories, this study clarified the relationship between lesion location on MRI and functional status, including cognitive aspects, in post-stroke patients. This machine learning-based approach demonstrates the potential to support early dysphagia assessment using routinely available data such as MRI and cFIM. In the future, combining imaging data with simpler and more targeted cognitive assessment tools may further enhance accessibility and accuracy, enabling broader application in diverse clinical environments.

## Data Availability

The datasets generated and/or analyzed in this study are available from the corresponding author upon reasonable request.
